# Bmal1 knockout aggravates *Porphyromonas gingivalis*-induced periodontitis by activating the NF-κB pathway

**DOI:** 10.1590/1678-7757-2024-0388

**Published:** 2025-02-21

**Authors:** Ye TIAN, Xinran LIU, Qiuyu LU, Jiaxin LI, Tianqi WANG, Mei TIAN, Yan DING, Jinle LI

**Affiliations:** 1 Sichuan University West China Hospital of Stomatology National Clinical Research Center for Oral Diseases Chengdu China Sichuan University, West China Hospital of Stomatology, National Clinical Research Center for Oral Diseases, State Key Laboratory of Oral Diseases, Department of Geriatric Stomatology, Chengdu, China.; 2 Sichuan University West China Hospital of Stomatology National Clinical Research Center for Oral Diseases Chengdu China Sichuan University, West China Hospital of Stomatology, National Clinical Research Center for Oral Diseases, State Key Laboratory of Oral Diseases, Chengdu, China.; 3 Sichuan University West China Healthcare Hospital of Sichuan University Chengdu China Sichuan University, West China Healthcare Hospital of Sichuan University, Chengdu, China.; 4 Sichuan University West China Hospital of Stomatology National Clinical Research Center for Oral Diseases Chengdu China Sichuan University, West China Hospital of Stomatology, National Clinical Research Center for Oral Diseases, State Key Laboratory of Oral Diseases, Department of General Clinic, Chengdu, China.

**Keywords:** Circadian clock, Periodontitis, BMAL1, NF-κB, p65

## Abstract

Circadian rhythm disorders and NF-κB are closely linked and can exacerbate periodontitis. However, the mechanisms via which circadian rhythm-related genes influence periodontitis are not yet fully understood.

**Objective:**

We investigated the effect of brain and muscle Arnt-like protein-1 (BMAL1) on the NF-κB pathway and downstream inflammatory factors on periodontitis. In this study, Bmal1 homozygous knockout and periodontitis mouse models were established.

**Methodology:**

Bone marrow-derived macrophages (BMDMs) from *Bmal1*^-^/^-^ mice were cultured and stimulated with lipopolysaccharides. Bone resorption was detected using micro-computed tomography and histological analyses. Gene and cytokine expression was assessed using quantitative reverse-transcription PCR and ELISA. The nuclear translocation of p65 was detected using immunofluorescence.

**Results:**

Our findings indicate that Bmal1 knockout exacerbates periodontitis severity in mice by activating the NF-κB signaling pathway with increased nuclear translocation of p65 (*p*<0.05), as well as increased expression of Il-1b, Il-6, and Tnfα (*p*<0.01), along with decreased Nr1d1 expression (*p*<0.05) in BMDMs under inflammation.

**Conclusion:**

The results highlight the protective role of Bmal1 in periodontitis and suggest its potential link to the circadian clock's influence on the disease.

## Introduction

Circadian rhythms are the structural basis for molecular clocks.^1^ A 24-hours cycle of the life rhythm (biological clock) influences individual life events in time with the alternations between day and night;^2,3^ it is also closely related to human health.^4^ In mammals, the suprachiasmatic nucleus of the hypothalamus is considered the central circadian clock.^5^ This clock detects changes in light levels via specialized photoreceptors, enabling it to synchronize the internal rhythms of the body and the external environment. Circadian clock genes are expressed in the peripheral tissues, such as heart, liver, kidney, fibroblasts, and lymphocytes.^6^ The periodic oscillating expression of these genes is regulated by a family of transcription factors called “clock genes,” of which the brain and muscle Arnt-like protein-1 (BMAL1) plays a critical role as the promoter of the whole biological clock system.^7^ Dysregulation of the expression of the circadian clock gene may lead to various pathological conditions, including autoimmune diseases, insomnia, seasonal depression, Alzheimer’s disease, and cancer.^8^ Current research shows that circadian clock genes are closely related to immune and inflammatory responses and affect bone metabolism.^9^

Periodontitis, an inflammatory disease of the periodontal support tissues, is accompanied by bone resorption. Although the relation between circadian clock genes and periodontitis has not yet been clarified, circadian clock genes are regularly expressed in gingival fibroblasts and periodontal membrane cells,^10^ and their functions are regulated by the circadian clock.^11^ Clock genes also have been proven to significantly affect bone metabolism, such as by regulating osteoclast activity by their interaction with family members of steroid receptor coactivator.^12^ Recent studies have demonstrated circadian rhythm disorders can worsen periodontitis via Bmal1,^13^ but Melatonin supplementation in patients with periodontitis significantly improved essential periodontal parameters such as pocket depth and clinical attachment loss.^14^ The NF-κB signaling pathway, which plays a key role in inflammation, has been shown to participate in the inflammatory processes of periodontitis.^15,16^Additionally, it is directly regulated by the circadian clock, which modulates immune responses to infectious agents largely by activating the NF-κB signaling pathway.^13,17^

We hypothesized circadian clock genes, including *Bmal1*, may be involved in the occurrence and development of periodontitis. Therefore, we established a periodontitis model of *Bmal1*^-^mice to explore the regulatory effect of *Bmal1* on the NF-κB pathway and its downstream inflammatory factors in the progression of periodontitis.

## Methodology

### Animal model establishment

To minimize pain in the experimental animals, we reduced the number of animals required for the study and utilized effective anesthetic and antalgic measures. Four-week-old *Bmal1*^-^ C57BL/6 mice were purchased from the Nanjing Jiancheng Bioengineering Institute and housed in the Experimental Animal Center of Science and Technology Park of Sichuan University. The Experimental Animal Management Committee of Sichuan Province approved all procedures in accordance with the ARRIVE guidelines (WCHSIRB-D-2017-266). After a breeding period, eight-week-old females of *Bmal1*^-^ and *Bmal1*^+/+^ offspring were chosen as experimental animals, with seven mice in each group. Inclusion criteria: the animals had to be either *Bmal1*^-^ (homozygous) or *Bmal1*^+/+^ (wild-type), confirmed by genotyping the DNA extracted from toe and tail samples and they needed to be healthy, with no obvious diseases. Exclusion criteria: animals that did not properly undergo the bacterial inoculation or those that may not have responded to the model induction (e.g., failure to develop periodontitis) and animals that did not undergo the whole experimental process. The mice were separated into four groups: (1) control group (*Bmal1*^+/+^) with bacterial infection, (2) control group (*Bmal1*^-^) without bacterial infection, (3) periodontitis group (*Bmal1*^+/+^) with bacterial infection, and (4) periodontitis group (*Bmal1*^-^) without bacterial infection. After being numbered with random number generation tools, *Bmal1*^+/+^ and *Bmal1*^-^ animals were randomly assigned to the groups. All experiments were conducted independently in triplicate. Experiments were conducted in triplicate on three separate occasions, and *N*=7 for each group, resulting in a total sample size of *N*=21 for each group. All mice were fed standard food and water at the State Key Laboratory for Oral Disease.

### Animal breeding

The mice were separated by sex to acclimate to their environment. When the estrus cycle of the female mice was observed by the age of eight weeks, *Bmal1*^-^ mice were put in a cage in a 1:2 male-to-female ratio until enough wild-type (*Bmal1*^*+/+*^) and homozygous (*Bmal1*^-^) mice were available for the experiments. The genotypes of the newborn mice were characterized three to four weeks after birth. All mice were housed at 20–25 °C with 65–69% humidity, maintained in a 12-hours light/dark cycle, and given free access to water and food. The protocol was aimed to minimize harm to the animals.

### DNA extraction and genotyping

DNA extraction from toe and tail samples and subsequent genotyping were performed on all experimental animals. Mice were fixed and numbered with clipped toes. The toes and approximately 2-mm-long tail samples were inserted into an EP tube using eye tweezers. The wounds were pressed with sterilized cotton balls to stop bleeding. All samples were stored in a refrigerator at -20 °C until further analysis. A NaOH-EDTA solution (75 μL, 25 mM NaOH + 2 mM EDTA) (Beyotime, Shanghai, China) was added to each tube, the tubes were heated in a thermocycler (Thermo Fisher Scientific, USA) at 96 °C for 60 min, and then placed on ice. Next, 75 μL of 40 mM Tris-HCl (pH 8.0) (Beyotime) was added to each tube, which was vortex-mixed at 4,000 rpm for three min. The PCR products were separated using 2% agarose gel electrophoresis (Beyotime) and stained with Goldview (Solarbio, China). The target band was 399 bp for the wild-type mice, 600 bp for the homozygous mice, and 600 and 399 bp for the heterozygous mice. The primer sequences utilized for quantitative reverse-transcription PCR (qRT-PCR) detection are shown in Supplementary Table 1. The mice were marked as wild-type or homozygous according to the genotyping results (Supplementary Figure 1).

**Figure 1:**
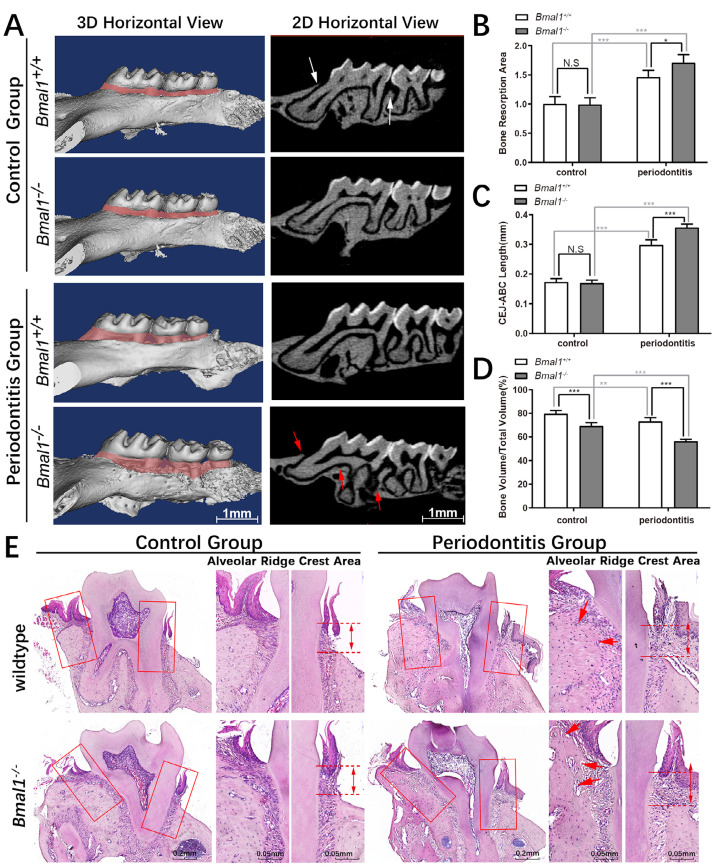
Bmal1 knockout aggravates bone resorption in the periodontitis group. (a) Bone resorption of the maxilla in each group. Three-dimensional (3D) reconstruction and two-dimensional (2D) CT images are shown. (b) Quantitative analysis of the alveolar bone resorption area. (c) Quantitative analysis of the mean distance from the CEJ to the ABC in each group. (d) Quantitative analysis of bone volume fraction in the first and second molar area. (e) Histological analyses using hematoxylin and eosin staining to identify attachment loss. *p<0.05, **p<0.01, ***p<0.001, N.S.: No statistically significant difference. ABC, alveolar bone crest; CEJ, cementoenamel junction; CT, computed tomography

### Bacterial culture

*Porphyromonas gingivalis* (ATCC 33277, USA) was used as infectious agent. Briefly, revived strains were inoculated on sheep blood plates (FisherBrand, USA) (475 mL ddH_2_O, 15 g Trypticase Soy Broth, 5 mL Hemin, 50 µL vitamin K, and 7.5 g agar; 500 mL final volume) using the multistage inoculation method, incubated in an anaerobic chamber with constant temperature of 37 °C (Gene Science, USA) for three to four days, and the strain growth was observed. Individual colonies were selected and inoculated into Brain Heart Infusion (BHI) liquid medium (Oxoid, England) (199 mL ddH_2_O, 7.4 g Brain Heart Infusion medium, 1 mL Hemin, 0.1 mg vitamin K; 200 mL final volume) for the amplification culture, in which each 5-mL culture was cultivated for three to four days. After three to four days, the cultures were transferred to 50 mL medium and centrifuged at 7,000 rpm at 4 °C for 30 min using a cryogenic ultra-high-speed centrifuge (Thermo Fisher Scientific). The medium was discarded, 1× phosphate-buffered saline (PBS) was added for re-suspension, and carboxymethylcellulose (CMC) was added to prepare a bacterial suspension with a CMC mass fraction of 2%. The number of bacteria was within 10^9^–10^10^ CFU/mL.

### Establishment of a periodontitis model

All animals were treated with kanamycin (0.5 mg/mL; J&K Scientific, China) for three days to clear the oral bacteria, followed by three days without antibiotic treatment. On the seventh day, oral bacterial implantation was performed for four consecutive days. Briefly, a small dental brush was dipped in the bacterial solution, and the surfaces of the upper and lower buccal and palatal periodontal tissues of the mice were coated. After each coating, the mice were prohibited from drinking water for two hours. After the completion of the initial four-days coating, a second round of coating was conducted for four days, with a two days interval. Samples were collected 45 days after coating.

### Sample preparation

After establishing the experimental animal model, the mice in each group were anesthetized with chloral hydrate and sacrificed via cervical dislocation. All the animals were included in the subsequent analysis. The tissues of the molar area in the maxillary and mandible bones were trimmed, collected, and soaked in RNAlater^®^ (Invitrogen, USA) at 4 °C for RNA. Periodontal samples were placed into pre-cooled tubes containing beads (Nextadvance Company, USA) and homogenized using a Bullet Blender® (Nextadvance Company, USA) at speed “5” for two five-minute cycles. The resulting tissue fragments were suspended in 1 ml of lysis buffer and incubated at 4°C for one hour. Following centrifugation at 12,000 rpm for 15 minutes, the supernatant was collected and stored at -80°C for subsequent analysis. The trimming range preserved the alveolar bone tissue in the molar area of the jaw, and the dental crown was removed with a surgical blade. After removing the skin tissue, the bilateral maxilla and skull were soaked overnight in 4% paraformaldehyde. After continuous washing for eight hours, the bilateral maxilla was separated, and the alveolar bone containing the first to third molars was kept intact. The central incisor was removed and stored in 70% alcohol at 4°C for histomorphologic examination.

### Micro-computed tomography (μCT)

The maxillary samples were appropriately trimmed before performing the µCT scans. Briefly, the samples were placed to keep the teeth parallel to the scanning rays. Scanning was performed using a µCT instrument (Scanco Medical, Switzerland) at a 12 μm voxel size, 55 kV, 145 μA, and 300 ms integration time. The parameter settings for scanning and analyses were set as previously published.^18^ After scanning, 3D reconstruction was performed using the Materialise Mimics software. Scanco Evaluation, Photoshop, and ImageJ software were utilized to assess the height and area of periodontal bone resorption. Additionally, the distance from the cementoenamel junction (CEJ) to the alveolar bone crest (ABC) was measured at six sites: the distal proximal surface of the first molar, three buccal cusps of the first molar, and two buccal cusps of the second molar.

### Histological staining

Following μCT imaging, the maxillary samples were immersed in an EDTA decalcifying fluid at room temperature. After decalcification, the tissues were fixed in paraffin and sectioned at a sagittal orientation. For Hematoxylin-eosin staining, the slides underwent deparaffinization and hydration using xylenes and a series of graded alcohols. Subsequently, they were stained with hematoxylin solution, followed by rinsing, and then the slides were stained with an eosin solution.

### qRT-PCR

The alveolar bone in the jaw was trimmed, and the total RNA was extracted using an extraction kit of RNA (TaKaRa, Japan). cDNA was reverse-transcribed using a first-strand synthesis kit of cDNA (Thermo Fisher Scientific) following the manufacturer’s instructions. PCR amplification and mRNA expression analysis of the target genes were performed using a TaKaRa TB Green Premix Ex Taq II (Tli RNase H Plus, TaKaRa, Japan). The qRT-PCR primer sequences are shown in Supplementary Table 2.

### Protein extraction for an ELISA

An ELISA was conducted to measure cytokine levels in the extracts using commercially available kits: TNF-α, IL-6, and IL-1β (eBioscience, USA). The results were reported in pg/ml.

### Primary bone marrow-derived macrophage (BMDM) culture

The mice’s bilateral femur and tibia were separated, washed in sterile PBS, and then transferred to an ultra-clean table to strip the muscle fascia and cartilage. The metaphysis was then exposed, the bilateral ends were removed with ophthalmic scissors, and the bone marrow was rinsed with Dulbecco’s Modified Eagle Medium (DMEM; Hyclone, USA) using a 1-mL syringe. The medium with bone marrow was centrifuged at 500 rpm for five minutes, and the isolated cells were suspended in a red cell lysis solution. The cells were centrifuged again for five minutes at 4 °C and then washed with PBS. Then, 20 ng/mL macrophage colony-stimulating factor (Sigma, USA), 20% FBS with 1% double antibody, and high-glucose DMEM were added. After three days, the spent medium was changed, and mature bone marrow macrophages were obtained via passage to p1 on the following two days. Lipopolysaccharide (LPS; 100 ng/mL) was added to the experimental group, whereas the control group was not treated. After 24 hours, samples were collected and stored in RNAlater^®^ at 4°C for RNA extraction and qRT-PCR detection. For characterization of BMDMs, Lipopolysaccharide (LPS; 10ng/mL) and IL-13 (50ng/mL) was added to experimental group as described.^19^

### Statistical analysis

The experimental data were showed as the means ± standard deviations of independent samples and statistically analyzed using GraphPad Prism 8.0 software. Each experiment included groups of seven mice with a similar distribution of males and females, with one sample taken from each mouse. For parametric data (bone resorption area, CEJ-ABC length, mRNA expression), differences between groups were analyzed using one-way analysis of variance (ANOVA), whereas the Mann–Whitney U test was employed for non-parametric data (the percentage of positive cell, integrated density/area). A p-value of less than 0.05 was considered statistically significant.

### Blinding

Blinding method was used for researchers who handle the animals, to manage treatments, or analyze outcomes.

## Results

### Bone resorption and attachment loss are increased in Bmal1-knockout mice with periodontitis

Micro-CT scans were performed on the maxilla of the four groups of mice, and the results were reconstructed using 3D software. The region between the ABC and CEJ of the maxillary first molars and the third molars are shown. The range of the alveolar bone absorption region was evaluated by analyzing the CT and buccal images (Figure 1a). Two-dimensional CT images in the direction of the crown root showed the alveolar bones of the *Bmal1*^-^ and wild-type control mice were continuous and smooth. The crests of the alveolar ridges were sharp, and the proximal-distal alveolar ridges of the first molars were not clearly absorbed (Figure 1a). In the wild-type periodontitis group, the bone in the root furcation of the first molar was rough and discontinuous, showing pitted absorption (Figure 1a). The proximal and distal alveolar ridges were absorbed, and the alveolar crest was round and blunt. Bone absorption occurred in the *Bmal1*^-^ periodontitis group. In this group, the proximal and distal alveolar ridge absorption of the first molar was evident, along with pitted absorption. We observed no noticeable alveolar bone resorption in the *Bmal1*^-^ control and wild-type groups (i.e., *Bmal1*^*+/+*^ control group), whereas resorption was observed in the two periodontitis groups. Bone loss was prominent in the *Bmal1*^-^ periodontitis group; the range of resorption was significantly larger compared with that in the wild-type periodontitis group, and the alveolar crest was destroyed (Figure 1a). The TRAP stain showed similar result considering the osteoclasts activation that the osteoclasts significantly increased in the *Bmal1*^-^ periodontitis group (Supplemental Figure 2a,b).

Quantitative statistical analysis of the alveolar bone resorption area showed no significant difference in the degree of resorption between the two control groups (*p*>0.05); however, the extent of resorption in the wild-type control (*Bmal1*^*+/+*^ control group), which were treated with oral bacteria, displayed a significant increase. Similarly, bone loss in the wild-type (*Bmal1*^*+/+*^) periodontitis group was significantly more severe than in the uninfected wild-type (*Bmal1*^*+/+*^) group (*p*<0.001). The extent of bone resorption in the *Bmal1*^-^ periodontitis group was also significantly higher than in the *Bmal1*^-^ control group (*p*<0.001). Moreover, alveolar bone resorption in the *Bmal1*^-^ periodontitis group was higher than in the wild-type periodontitis group (*p*<0.05) (Figure 1b).

Measurements revealed no significant difference in the mean distance of the CEJ-ABC region between the two control groups, whereas the mean distance increased in the periodontitis groups. Bone resorption in the *Bmal1*^-^ periodontitis group was significantly greater than that in the wild-type periodontitis group (*p*<0.01), suggesting the degree of vertical bone resorption in *Bmal1*^-^ mice during periodontitis was higher than in wild-type mice (Figure 1c).

The results also revealed the bone volume/total volume (BV/TV) ratio in the *Bmal1*^-^ control group was lower than in the wild-type group (*p*<0.01); similarly, the BV/TV ratio was significantly decreased in the *Bmal1*^-^ periodontitis group compared with that in the wild-type periodontitis group (*p*<0.001). Additionally, we observed a significantly decreased BV/TV ratio between the two *Bmal1*^-^ groups (*p*<0.01), which was greater than that between the wild-type groups (*p*<0.01) (Figure 1d).

No attachment loss was observed in the palatal periodontal tissues of both groups without bacteria (CEJ-ABC). However, the distance increased in the two groups after bacterial infection and was even greater in the *Bmal1*^-^ group. The alveolar bones of the control groups (wild-type and *Bmal1*^-^) were intact, the cortical surface of the bone was smooth, and no apparent bone resorption sites were observed. In contrast, bone resorption was observed in the buccal alveolar bone in both the wild-type and *Bmal1*^-^ periodontitis groups but was more pronounced in the *Bmal1*^-^ group (Figure 1e).

### Bmal1 knockout increased NF-κB and p65 expression in inflamed periodontal tissues

**Figure 2:**
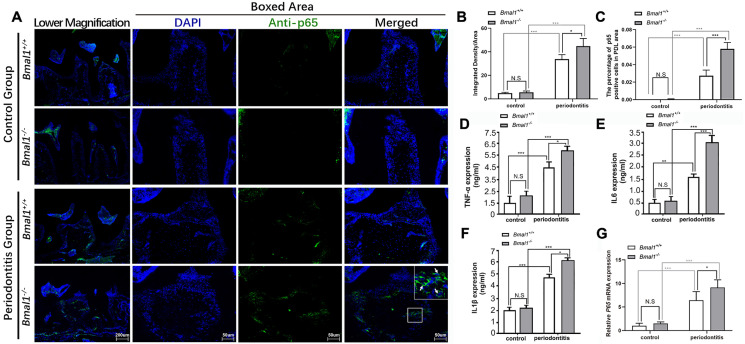
Bmal1 knockout increases p65 expression in periodontitis. (a) Immunofluorescence staining of p65 in the maxilla. Green fluorescence shows the expression of p65. (b) Statistical analysis of the integrated density/area of p65-positive cells in (a). (c) Statistical analysis of the percentage of p65-positive cells in (a). (d-f) ELISA was used to detect the protein expression levels of TNFα, IL1β and IL6. (g) qRT-PCR was used to detect the mRNA expression levels of p65. PDL: periodontal ligament; DAPI: nuclear dye, blue. White arrows show p65-positive cells. *p<0.05, ***p<0.001, N.S.: No statistically significant difference. ELISA: enzyme-linked immunosorbent assay. qRT-PCR: Quantitative reverse-transcription polymerase chain reaction

To study the differences in the inflammatory response in the periodontal tissues of mice in each group, immunofluorescence and qRT-PCR were performed to detect the expression of p65, a key factor in the inflammation-related NF-κB signaling pathway. p65 entry was observed via its colocalization with the nucleus, indicating the activation of the NF-κB pathway. No significant differences were observed in p65 expression in the periodontal membrane of the wild-type and *Bmal1*^-^ control groups. The expression level of p65 in the periodontal membrane in the two periodontitis groups was significantly increased and nuclear translocation was observed. The expression of p65 in the *Bmal1*^-^ periodontitis group was higher than in the wild-type periodontitis group, and the number of p65-positive cells increased (Figure 2a). Quantitative analysis of the average fluorescence intensity and p65-positive cell proportion showed the average optical density and intracellular fluorescence intensity were enhanced in the *Bmal1*^-^ periodontitis group and the ratio of p65-positive cells increased (Figure 2b, 2c). The ELISA results showed the NF-κB signaling related inflammatory cytokines such as TNFa, IL-1β, and IL-6 increased in the *Bmal1*^-^ periodontitis group (Figure 2d-f). The qRT-PCR results showed the mRNA expression of *p65* was similar between the wild-type control and *Bmal1*^-^ control groups. However, its expression in the periodontitis group significantly increased. Furthermore, the expression of *p65* in the periodontal tissues of the *Bmal1*^-^ periodontitis group was higher than the wild-type periodontitis group (Figure 2g).

### Bmal1 knockout upregulated the activation of the NF-κB pathway in LPS-stimulated BMDMs

**Figure 3:**
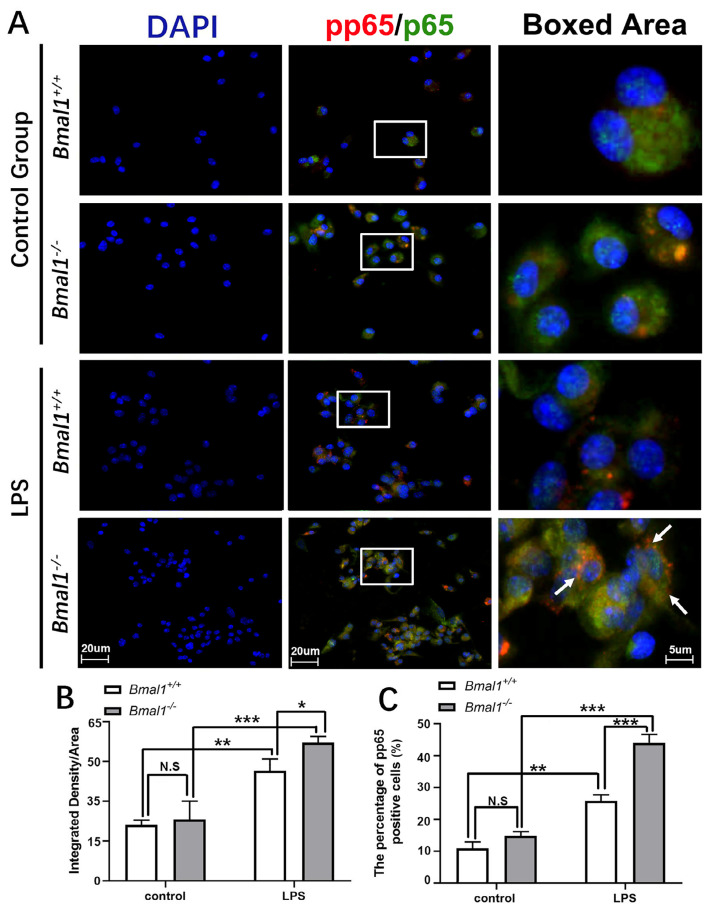
Bmal1 knockout increases pp65 and p65 expression in LPS-stimulated BMDMs. (a) pp65 and p65 immunofluorescence staining of bone marrow macrophages. White arrows show pp65 and p65-positive cells. (b) Average optical density. (c) Percentage of pp65-positive cells. *p<0.05, **p<0.01, ***p<0.001, N.S.: No statistically significant difference. BMDM, bone marrow-derived macrophage; LPS, lipopolysaccharide

Immunofluorescence staining was also performed on BMDMs. The number of pp65 and p65-positive cells in the LPS-stimulated *Bmal1*^-^ group was significantly higher than in the wild-type LPS-stimulated group (Figure 3a). No nuclear translocation of p65 was observed in the control groups, whereas cells with the nuclear translocation of p65 were observed in the LPS-stimulated groups. Quantitative analysis of the average fluorescence intensity and p65-positive cell proportion showed that pp65 and p65 expression was enhanced in the LPS-stimulated group, with the *Bmal1*^-^LPS-stimulated group showing a significant increase compared with that in the wild-type LPS-stimulated group (Figure 3b, 3c).

### Bmal1 knockout increased the expression of inflammatory genes in periodontal inflammatory tissues and BMDMs stimulated by inflammation

**Figure 4:**
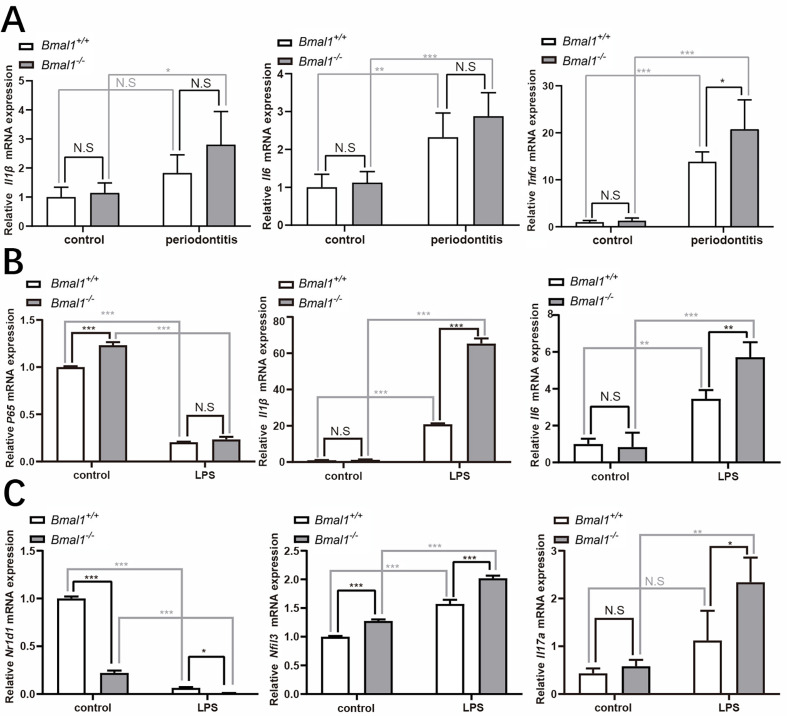
Bmal1 knockout affects inflammatory factor expression in periodontitis and LPS-stimulated BMDMs. (a) Expression levels of Il1b, Il6, and Tnfa. (b) mRNA expression of inflammatory cytokines in the bone marrow macrophages of Bmal1-knockout mice. (c) mRNA expression of N1rd1, Nfil3, and Il17a. *p<0.05, **p<0.01, ***p<0.001, N.S.: No statistically significant difference. BMDM, bone marrow-derived macrophage; LPS, lipopolysaccharide

We also performed *in vitro* and *in vivo* experiments to determine whether *Bmal1* knockout influences the immune response (Figure 4). Although *Il1b* and *Il6* expression showed no significant difference between *Bmal1*^*+/+*^ and *Bmal1*^-^ mice in the periodontitis group, an increasing trend was noticed (Figure 4a). *Tnfa* expression increased in the *Bmal1*^-^ with periodontitis group compared with *Bmal1*^*+/+*^ with periodontitis group (Figure 4a). The *in vitro* results showed *p65* expression was not different between the *Bmal1*^*+/+*^ and *Bmal1*^-^ LPS-stimulated groups (Figure 4b). However, the expression levels of *Il1b* and *Il6* increased in the *Bmal1*^-^ LPS-stimulated group (Figure 4b). The cauterization of BMDMs showed that under stimulation the expression of Arg1 and IL-10 of BMDMs significantly increased (Supplemental Figure 2c). In addition, we also detected the expression levels of *Nr1d1* and *Nfil3* in the primary bone marrow macrophages of *Bmal1*^-^ mice after LPS stimulation. Following *Bmal1* gene knockout, the expression level of *Nr1d1* decreased, whereas the expression level of *Nfil3,* which is inhibited by Bmal1, increased. No differences in the expression of *Il17a* were observed in the *Bmal1*^*+/+*^ and *Bmal1*^-^ control groups. However, under an inflammatory environment, the *Bmal1*^-^ group showed a significant increase in *Il17a* expression (Figure 4c).

## Discussion

The circadian rhythm plays a role in the homeostasis of the immune system. When this rhythm is disrupted, inflammatory responses may be activated.^20^ Several chronic inflammatory diseases are related to circadian clock genes.^21^ We observed bone resorption and attachment loss were more severe after *Bmal1* knockout. However, the underlying mechanism remains unclear.

NF-κB plays an essential role in periodontitis^22^ and *Bmal1,* and NF-κB interacts in regulating inflammatory responses.^23^ Therefore, we assessed the activity of the NF-κB pathway after *Bmal1* knockout *in vivo* and *in vitro*, focusing on p65, a component of the NF-κB family that mediates the transcription of several genes.^24^ qRT-PCR and immunofluorescence staining showed that knocking out *Bmal1* activated the NF-κB pathway and increased p65 expression in mice, thereby increasing the expression of inflammatory factors *in vivo* and *in vitro*. Furthermore, macrophages play a crucial role in the destruction and restoration of periodontitis via their immune activities.^25^ Notably, the nuclear translocation of p65 is a crucial component of the NF-κB pathway.^26^ In bone marrow macrophages, we confirmed that following LPS stimulation, *Bmal1*^-^ cells showed increased p65 nuclear localization and an upregulated expression of inflammatory factors, including IL-1β and IL-6. Therefore, we conclude that *Bmal1* suppresses NF-κB/p65 and regulates the expression of downstream inflammatory factors.

The downstream gene *Nr1d1* is another circadian clock gene linked with *Bmal1.*^27^ *Nfil3*, a transcriptional factor involved in many immune functions, is regulated by *Nr1d1.*^28^ Our study on BMDMs showed *Bmal1* knockout suppressed *Nr1d1* expression but increased *Nfil3* expression. IL-17a, a cytokine that mediates the development and progression of periodontitis,^29^ is reportedly inhibited by *Nr1d1.*^30^ We observed IL-17a expression increased in the *Bmal1*^-^ group under LPS stimulation, indicating *Bmal1* may regulate periodontitis via *Nr1d1*.

## Conclusions

In conclusion, we validated the vital role of Bmal1 in the development of periodontitis and demonstrated *Bmal1* knockout may promote bone destruction and inflammation during periodontitis through the NF-κB pathway. These findings underscore a new dimension of the influence of circadian rhythm on periodontitis progression, potentially paving the way for treatments centered on specific clock genes via chronotherapy, targeted therapies, and risk assessment strategies.
